# A Breach in Plant Defences: *Pseudomonas syringae* pv. *actinidiae* Targets Ethylene Signalling to Overcome *Actinidia chinensis* Pathogen Responses

**DOI:** 10.3390/ijms22094375

**Published:** 2021-04-22

**Authors:** Antonio Cellini, Irene Donati, Brian Farneti, Iuliia Khomenko, Giampaolo Buriani, Franco Biasioli, Simona M. Cristescu, Francesco Spinelli

**Affiliations:** 1 Department of Agricultural and Food Sciences, Alma Mater Studiorum—University of Bologna, 40127 Bologna, Italy; antonio.cellini2@unibo.it (A.C.); i.donati@unibo.it (I.D.); giampaolo.buriani2@unibo.it (G.B.); 2 Research and Innovation Centre, Fondazione Edmund Mach, 38010 San Michele all’Adige, Italy; brian.farneti@fmach.it (B.F.); iuliia.khomenko@fmach.it (I.K.); franco.biasioli@fmach.it (F.B.); 3 Life Science Trace Gas Facility, Radboud University, 6525 AJ Nijmegen, The Netherlands; s.cristescu@science.ru.nl

**Keywords:** bacterial canker of kiwifruit, plant hormones, plant immunity, stomata opening, virulence factors

## Abstract

Ethylene interacts with other plant hormones to modulate many aspects of plant metabolism, including defence and stomata regulation. Therefore, its manipulation may allow plant pathogens to overcome the host’s immune responses. This work investigates the role of ethylene as a virulence factor for *Pseudomonas syringae* pv. *actinidiae* (Psa), the aetiological agent of the bacterial canker of kiwifruit. The pandemic, highly virulent biovar of this pathogen produces ethylene, whereas the biovars isolated in Japan and Korea do not. Ethylene production is modulated in planta by light/dark cycle. Exogenous ethylene application stimulates bacterial virulence, and restricts or increases host colonisation if performed before or after inoculation, respectively. The deletion of a gene, unrelated to known bacterial biosynthetic pathways and putatively encoding for an oxidoreductase, abolishes ethylene production and reduces the pathogen growth rate in planta. Ethylene production by Psa may be a recently and independently evolved virulence trait in the arms race against the host. Plant- and pathogen-derived ethylene may concur in the activation/suppression of immune responses, in the chemotaxis toward a suitable entry point, or in the endophytic colonisation.

## 1. Introduction

The key steps in pathogen recognition and plant defence activation are strictly controlled by hormonal signals [1,2]. For instance, stomata closure, mediated by abscisic acid (ABA), is an early response to microbial molecular markers to prevent the invasion of potential pathogens into the apoplastic space [3]. Subsequently, more specific defences are triggered by salicylic acid (SA)-, ethylene- and jasmonic acid (JA)-dependent signalling pathways. SA elicits responses against biotrophic pathogens, while ethylene/JA-mediated defences are activated against necrotrophs. Due to the opposite lifestyles of their target pathogens, it is generally recognised that SA and ethylene/JA signalling pathways are mutually antagonistic [2]. However, this basic model is complicated by the existence of many points of interaction among the signal cascades. Ethylene synthesis, for instance, is induced by SA [4,5] in the early stages of the infection, but contributes to SA signal suppression in its subsequent interplay with JA [1].

Thus, pathogens may produce plant hormones [6,7] or toxins with a hormone-like effect [8] to hijack plant defence mechanisms. Several strains and pathovars of the broad host spectrum pathogen *Pseudomonas syringae* were reported to produce ethylene [9], the JA-isoleucine analogue coronatine [10,11,12], the ethylene- and ABA-inducing factor HopAB2_PtoDC3000_ [13,14], and the auxin-regulating factor AvrRpt2 [15]. All these products interfere with SA signalling, thus impairing plant defences [16] and acting as virulence factors.

*P. syringae* pv. *actinidiae* (Psa), responsible for the bacterial canker of kiwifruit, has emerged since 2008 as a pathogen threatening the kiwifruit industry worldwide [17,18,19]. Five biovars have been identified according to their geographical origin and genetic features. Among them, biovar 3 is the one raising the most serious phytosanitary concern, as it includes the highly virulent, pandemic strains [20,21,22]. Psa can survive asymptomatically for long periods in the phyllosphere [23], and host infection occurs via natural opening such as stomata, which are considered a major entry point for Psa [24,25]. Therefore, factors promoting stomata opening, such as coronatine and ethylene, may enhance Psa pathogenicity.

Psa strains belonging to biovar 2 produce coronatine [26], which, among other effects, contrasts stomata closure [3]. However, coronatine does not appear to be strictly required for virulence, since the highly virulent strains of biovar 3 do not synthesise this toxin [27].

Previous research demonstrated that ABA-mediated stomata closure can also be suppressed by ethylene [28,29]. In addition, ethylene may support pathogen growth and colonisation within the plant tissues, due to its antagonistic role against SA-mediated defences [30]. Ethylene is commonly produced by a wide range of microbes starting from two alternative precursors, 2-keto-4-methyl-thiobutyric acid (KMBA) [31,32] or 2-oxoglutarate [33,34]. The 2-oxoglutarate dioxygenase termed ethylene-forming enzyme (EFE) is required for the virulence of some *P. syringae* pathovars, such as *glycinea* [9].

Since ethylene is a key point of regulation, determining plant defence activation or suppression, its biosynthesis by Psa may contribute to the development of bacterial canker disease. To investigate ethylene-mediated crosstalk between host and pathogen, several details require consideration, including the timing of ethylene emission, and the characterisation of plant and pathogen responses relevant to early stages of infection. The aims of this work were: (i) to verify the production of ethylene by Psa strains, in particular those belonging to the highly virulent, pandemic biovar 3, (ii) to investigate the possible roles of ethylene in the host–pathogen interaction, and (iii) to elucidate the genetic base of ethylene production in Psa and the environmental factors affecting its emission. The information emerging from these experiments may shed light on aspects of plant–pathogen interaction transcending a simple gene-vs.-gene model and provide new targets for disease control.

## 2. Results

### 2.1. Ethylene Emission by Pseudomonas syringae pv. actinidiae

**Figure 1:**
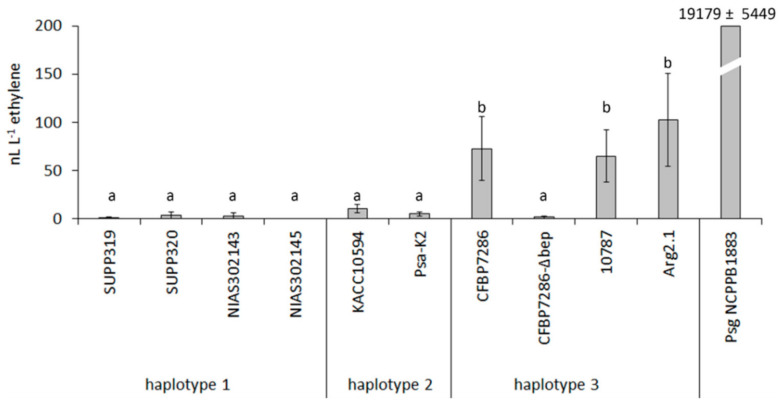
Ethylene emissions from bacterial cultures of several *Pseudomonas syringae* pv. *actinidiae* strains. Measures were taken 3 days after inoculation of fresh plant extract. Negative control (axenic medium) was taken for reference, and its reading was subtracted to all the values. Strain CFBP7286-Δ*bep* is a defective mutant for the gene Bacterial Ethylene Putative Producer (*bep*). Different letters indicate statistically different ethylene contents (*p* ≤ 0.05, *n* = 3) according to ANOVA and Fisher’s least significant difference test. A strain of *P. syringae* pv. *glycinea* (Psg NCPPB1883), used as the positive control, was not considered for statistics.

**Figure 2:**
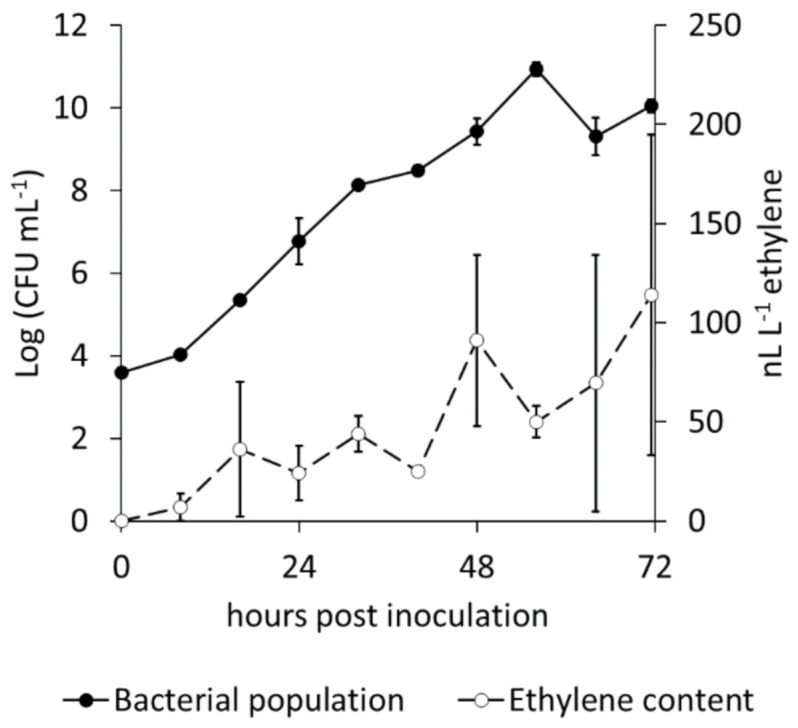
Comparative kinetics of population growth and ethylene release from *Pseudomonas syringae* pv. *actinidiae*. The strain CFBP7286 was grown in liquid culture. Values are presented as the average ± standard error (*n* = 4).

Ethylene emission by the different Psa biovars was studied both in artificial medium and fresh plant extract (FPE). In all these experiments, the emission baseline was determined from the respective non-inoculated medium. None of the Psa strains tested produced ethylene neither in rich (LB), nor minimal (modified CERIA 132) artificial medium. Axenic LB medium emitted ethylene up to 0.25 nL L^−1^ h^−1^ and this emission rate was not modified by bacterial growth. Among the Psa strains tested in this experiment, only those belonging to biovar 3 (CFBP7286, 10,787 and Arg2.1) showed a detectable ethylene release when grown on FPE (Figure 1). Boiling the FPE medium, although influencing the bacterial growth, did not impair ethylene production. Ethylene production by *P. syringae* pv. *glycinea*, that was used as positive control, was two orders of magnitude higher than by any Psa strain. Based on this result and on the availability of the annotated genome sequence, the strain CFBP7286 was selected for all the subsequent experiments. In this strain, the kinetics of ethylene accumulation in the headspace during Psa growth followed a linear (R^2^ = 0.7505) increase, while the bacterial population, as expected, grew exponentially (Figure 2). Thus, the highest bacterial density (10^9^ CFU mL^−1^) corresponded the peak of ethylene concentration (114 nL L^−1^).

**Figure 3:**
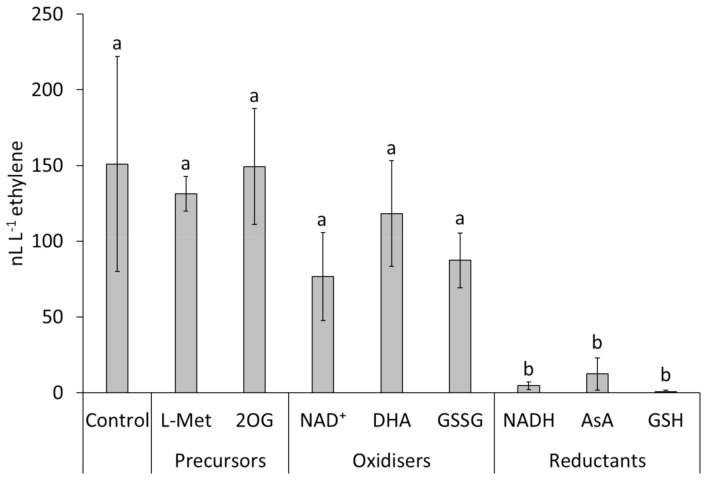
Ethylene emissions of *Pseudomonas syringae* pv. *actinidiae* strain CFBP7286. Measures were taken 72 h after inoculation in modified liquid media containing 1 mM L-methionine (L-Met), 10 mM 2-oxoglutarate (2OG), 2 mM NAD^+^, 2 mM dehydroascorbate (DHA), 1 mM oxidised glutathione (GSSG), 2 mM NADH, 2 mM ascorbic acid (AsA), or 2 mM glutathione (GSH). Different letters indicate significantly different (*p* ≤ 0.05, *n* = 3) ethylene emissions, according to ANOVA and Fisher’s least significant difference test.

Concerning the molecular basis of ethylene emission by Psa, the addition of L-methionine (1 and 0.01 mM) or 2-oxoglutarate (10 and 0.1 mM) to the growing medium did not increase ethylene production in any of the selected strains (Figure 3). The screening of the available Psa genomes (NCPPB3871, PA459 and CFBP7286) confirmed the absence of genes for the key enzymes of KMBA or EFE pathways. PCR analysis performed on the Psa strains listed in Table 1 further proved the absence of *efe* genes. Nonetheless, the comparison among the genomes of the biovar 3 (CFBP7286, ethylene-producing) and biovar 1 (NCPPB3871 and PA459) Psa strains highlighted the presence of several unique sequences. One of them, the contig OG6084 at locus KW9RS0106360 (NCBI reference NZ_AGNO01000059.1) has been classified as a putative oxidoreductase, which could be involved in the 2-oxoglutarate-dependend emission of ethylene. Indeed the sequence showed a 99% identity with Fe(II)/2-oxoglutarate hydroxylase of *P. syringae*. The presence of this gene, which was newly named as Bacterial Ethylene Putative Producer (*bep*), was tested by PCR in all Psa strains. PCR product corresponding to *bep* was found in all the Psa strains belonging to biovar 3, and in none of the others. To confirm the role of *bep* in ethylene formation, a site-specific mutagenesis was applied to Psa strain CFBP7286 to produce a *bep*-defective mutant (CFBP7286-Δ*bep*). The deletion of *bep* in CFBP7286 abolished ethylene emission in this strain (Figure 1). No significant differences in growth, motility or virulence were observed between the wild type and the mutant strain (Figure S1).

### 2.2. Modulation of Ethylene Emission in Pseudomonas syringae pv. actinidiae

Since ethylene emission in infected plants was recorded only at night (see Section 2.4), the bacterial sensitivity to light was tested by growing the cultures under continuous light or dark. Ethylene emission by bacterial cultures grown under light or dark did not show any significant difference, suggesting that light does not have a direct effect on the bacterial ethylene metabolism. The influence of the redox state of the growing medium was tested to verify a possible plant-mediated effect of light, since plant photosynthetic activity drives the reduction of soluble redox compounds, such as NAD(P)H, glutathione, ascorbate and thioredoxins. The presence of an oxidising factor, such as GSSG, NAD(P)^+^ or dehydroascorbate in the medium permitted ethylene emission, whereas reducing compounds, namely, GSH, NAD(P)H, ascorbate and dithiotreitol abolished it (Figure 3).

Finally, since ethylene was produced only when the pathogen was grown in FPE, but not in artificial medium, FPE solutes were separated into three fractions according to their molecular weight, to identify the size of the plant compound required for ethylene emission. No ethylene was emitted by cultures in minimal medium amended with FPE fractions of molecular weight above 10 kDa.

### 2.3. Effect of Ethylene on Bacterial Growth, Motility, Virulence and Host Plant Colonisation

**Figure 4:**
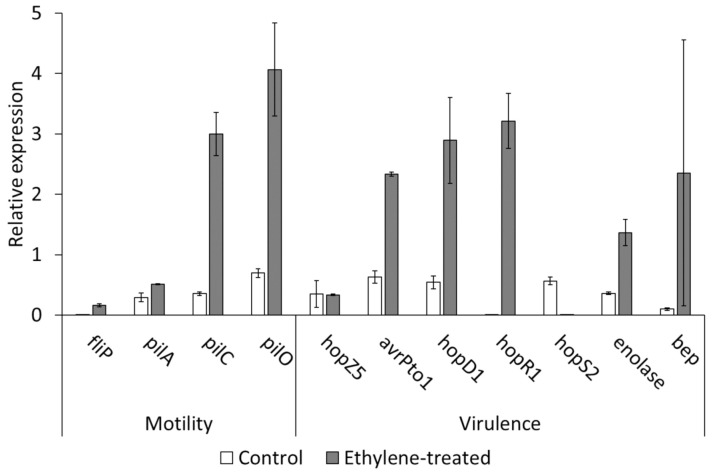
Expression of genes related to bacterial motility and virulence in liquid cultures of *Pseudomonas syringae* pv. *actinidiae* strain CFBP7286 exposed to ethylene. Data are shown as the relative expression compared to housekeeping genes. All data pairs, except *hopZ5* and *bep*, are significantly (*p* ≤ 0.05, *n* = 3) different according to Student’s T test.

Ethylene administration to Psa cultures did not influence the bacterial growth. However, 54% of the ethylene-exposed cultures showed a motility phenotype. In none of the controls was this phenotype observed. The expression of genes related to pilum formation (*pilC*, *pilO*) was stimulated by ethylene treatment on bacterial liquid cultures, compared to nontreated control (Figure 4). Exogenous ethylene treatments also increased the expression of virulence effectors (*avrPto1*, *hopD1*, *hopR1*). Despite a high variability in its expression, *bep* was also promoted by exogenous ethylene (Figure 4).

**Figure 5:**
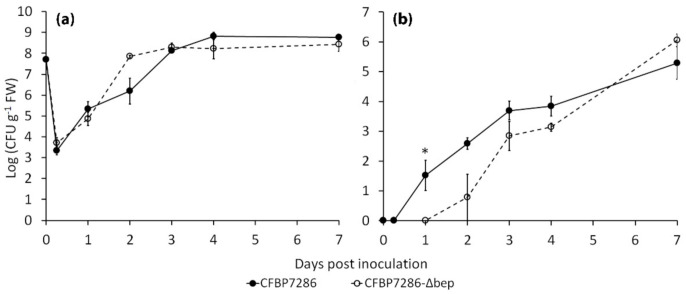
In planta growth of *Pseudomonas syringae* pv. *actinidiae*. (**a**) Epiphytic and (**b**) endophytic population of *Pseudomonas syringae* pv. *actinidiae* strains CFBP7286 and CFBP7286-Δ*bep* in in vitro *Actinidia deliciosa* plants was measured 1–7 days post inoculation. Values are presented as the average ± standard error (*n* = 3). An asterisk indicates a significant difference (determined by Student’s T test with *p* ≤ 0.05) in the relative data pair.

The role of bacterial ethylene in increasing pathogen aggressiveness and host plant colonisation was confirmed in planta. Compared to CFBP7286, the infection of *A. deliciosa* plants with Psa mutant (CFBP7286-Δ*bep*) impaired for ethylene production resulted in a lower endophytic population for the first 3 days after inoculation. However, the populations attained similar levels on day 7 (Figure 5). Epiphytic growth of CFBP7286-Δ*bep* was not affected, thus suggesting a role of ethylene in the invasion of the plant tissues, but not in the epiphytic fitness of the pathogen.

**Figure 6:**
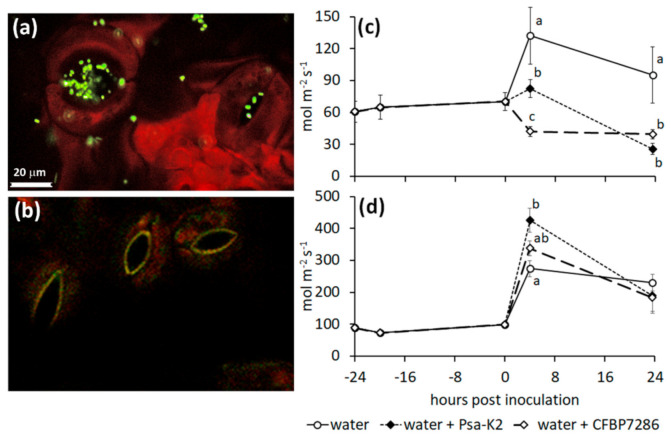
Stomata colonisation and stomata opening induced by *Pseudomonas syringae* pv. *actinidiae*. In vivo stomata colonisation (**a**,**b**) and stomatal conductance of *Actinidia deliciosa* (**c**) and *A. chinensis* (**d**) plants, after inoculation with *Pseudomonas syringae* pv. *actinidiae* strains CFBP7286 or Psa-K2. (**a**) In confocal laser scanning micrographs of *Actinidia deliciosa* stomata colonised by *Pseudomonas syringae* pv. *actinidiae* strains CFBP7286-GFPuv, each green rod is a single pathogen cell; (**b**) stomata in an uninfected leaf. (**c**,**d**) Stomatal conductance measured in real-time by gas exchange analyser (CIRAS-1). For each time point, significant differences (determined by ANOVA followed by Fisher’s least significant difference test with *p* ≤ 0.05, *n* = 4) are indicated by different letters.

Since stomata represent both the main entry points for endophytic colonisation and the first line of plant defence, the effect of inoculation with Psa on stomata opening was verified in *A. deliciosa* and *A. chinensis*. For the experiment, strains of Psa belonging to biovar 1 (SUPP319, producing neither coronatine nor ethylene), biovar 2 (Psa-K2, producing coronatine but not ethylene) and biovar 3 (CFBP7286, producing ethylene but not coronatine) were used. As the strain SUPP319 showed no effects on stomata in the first trials on *A. chinensis* (Figure S2), it was excluded from subsequent experiments. After an initial phase of epiphytic growth, occurring between 3 and 6 h after inoculation, Psa strain CFBP7286 colonised the open stomata (Figure 6a,b). In all the experiments, mock-inoculated (i.e., water-sprayed) plants increased their stomatal conductance after the spray. In contrast, no stomatal opening was observed after inoculation of *A. deliciosa* plants with either Psa strain. Rather, a reduced stomatal conductance was observed 4 (for CFBP7286) or 24 (for Psa-K2) hours post inoculation (Figure 6c). A different behaviour was observed in *A. chinensis* plants, where Psa-K2 elicited a significantly increased stomatal opening shortly after inoculation (Figure 6d). In all the cases where an increase in stomatal conductance was induced by bacterial inoculation, the effect was limited to 4 h post inoculation, and decreased thereafter.

**Figure 7:**
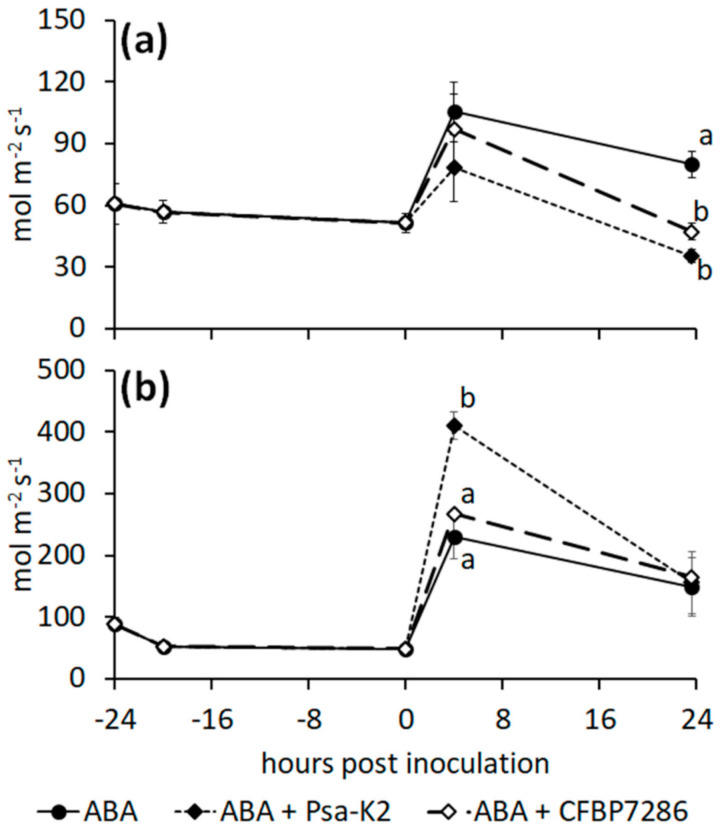
Interaction of *Pseudomonas syringae* pv. *actinidiae* with ABA on stomata. (**a**) Stomatal conductance of *Actinidia deliciosa* or (**b**) *A. chinensis* plants treated with ABA 1 day before inoculation with *Pseudomonas syringae* pv. *actinidiae* strains CFBP7286 or Psa-K2. For each time point, significant differences (determined by ANOVA followed by Fisher’s least significant difference test with *p* ≤ 0.05, *n* = 4) are indicated by different letters.

A 0.5 mM ABA treatment on *A. deliciosa* delayed CFBP7286-, but not Psa-K2-induced stomata closure (Figure 7a). In *A. chinensis*, ABA application abolished the stomata opening induced by the ethylene producing strain, CFBP7286, whereas the coronatine-producing strain, Psa-K2, retained the ability to induce stomata opening (Figure 7b). Comparable results were obtained by the direct measuring of stomata opening under CLS microscopy (Figure S3). Moreover, in *A. chinensis*, the application of ABA, abolishing the stomata opening induced by CFBP7286, reduced its endophytic population.

### 2.4. Ethylene Emissions from Infected Plants

**Figure 8:**
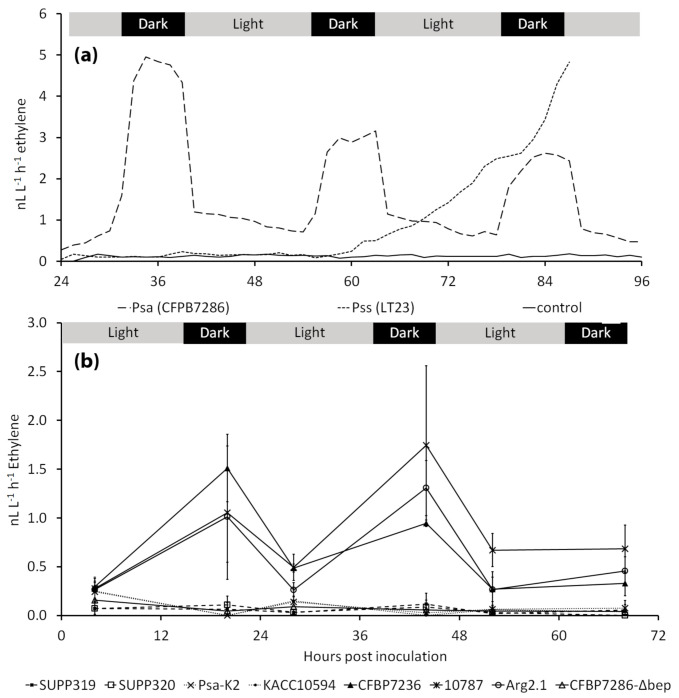
Time-dependent ethylene release from infected plants. (**a**) Ethylene release from in vitro *Actinidia deliciosa* ‘Hayward’ plants, inoculated with *Pseudomonas syringae* pv. *actinidiae* (Psa strain CFBP7286) or the compatible pathogen *Pseudomonas syringae* pv. *syringae* (Pss strain LT23). Data show the typical emission of singular samples. (**b**) Ethylene release from in vitro *Actinidia deliciosa* ‘Hayward’ plants, inoculated with several Psa strains belonging to biovars 1, 2 and 3. Values are presented as the average ± standard error (*n* = 3). In both experiments, the light:dark cycle was 16:8 h.

Real-time ethylene emission from CFBP7286-inoculated *A. deliciosa* plants is shown in Figure 8. The emission of ethylene required approximately 20–24 h from inoculation to be observed and was limited to the dark phase of the photoperiod. Plants release ethylene starting from 60 h after inoculation with an *Actinidia*-pathogenic *P. syringae* pv. *syringae* strain, but no diel pattern was observed in this case (Figure 8a). Ethylene emission was induced only by the ethylene producing strains belonging to biovar 3 (CFBP7286, 10,787 and Arg2.1), whereas plants inoculated with the ethylene deficient strain CFBP7286-Δ*bep* did not emit ethylene (Figure 8b). At the end of the experiment, 5 days after inoculation, no differences in endophytic bacterial populations were observed among plants inoculated with different Psa strains (data not shown).

**Figure 9:**
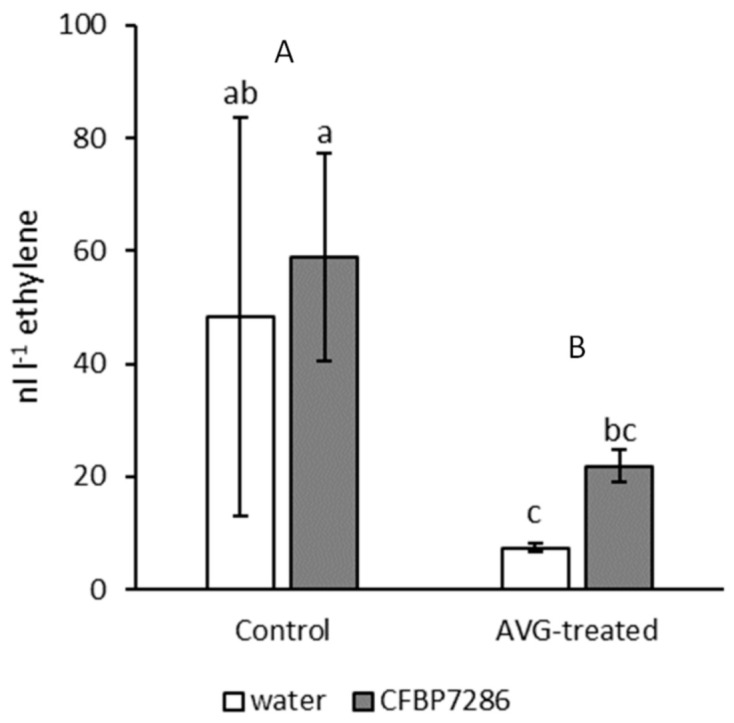
Ethylene emission from *Actinidia deliciosa* microcuttings, included in 500-mL jars with AVG-containing medium, 1 week after inoculation with *Pseudomonas syringae* pv. *actinidiae* strain CFBP7286. Different lower-case letters indicate significant differences (determined by two-way ANOVA followed by Fisher’s least significant difference test with *p* ≤ 0.05, *n* = 3) due to interaction between AVG treatment and pathogen inoculation. Upper-case letters refer to single-factor differences between AVG treatment and water.

The inclusion of aminoethoxyvinylglycyne (AVG) in the plant medium reduced by over 60% the release of ethylene from CFBP7286-inoculated samples, suggesting that the plant primarily contributed to the observed ethylene emission (Figure 9).

**Figure 10:**
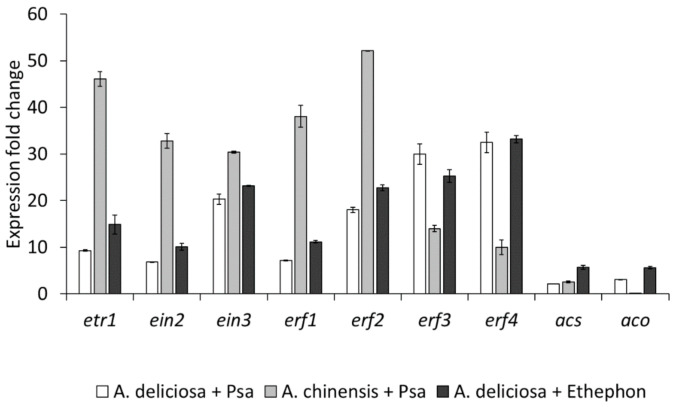
Expression of genes related to ethylene signalling in in vitro *Actinidia deliciosa* and *A. chinensis* plants. Gene expression was measured 6 h after inoculation with *Pseudomonas syringae* pv. *actinidiae* strain CFBP7286, or after treatment with ethylene. Data are expressed as fold change of relative gene expression, calculated with relation to housekeeping genes, compared to untreated plants of the same species. Values are presented as the average ± standard error (*n* = 3).

The infection with the ethylene-producing strain CFBP7386 induced in the host plant the expression of genes related to ethylene signal cascade (Figure 10). In contrast, the genes for ethylene production (*acs* and *aco*) were poorly induced by inoculation. Psa-induced gene expression was comparable to the one induced by ethylene treatment. Finally, the expression of genes related to ethylene signal cascade in *A. chinensis* was generally higher than in *A. deliciosa*.

### 2.5. Effect of Ethylene on the Host–Pathogen Interactions

**Figure 11:**
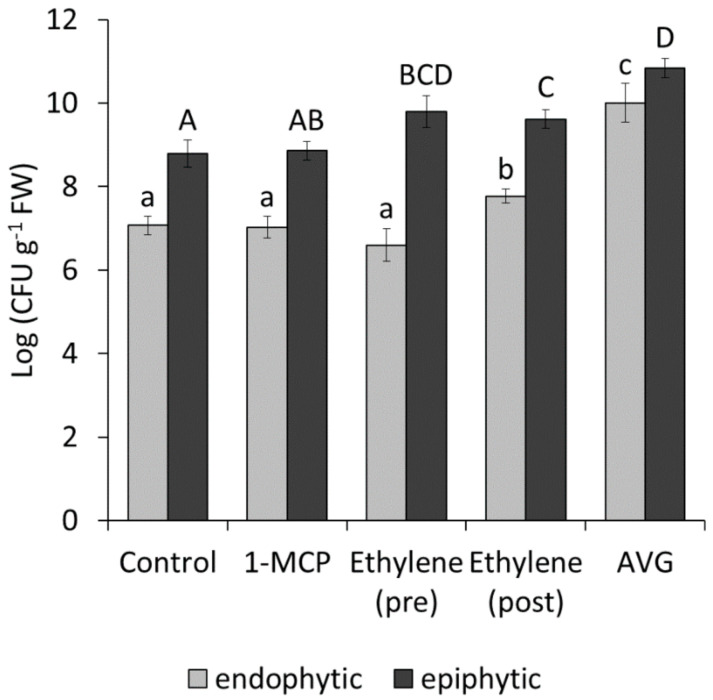
Effect of modulators of ethylene metabolim on bacterial colonisation. Endophytic and epiphytic populations of *Pseudomonas syringae* pv. *actinidiae* strain CFBP7286-GFPuv were measured in in vitro *Actinidia deliciosa* plants treated with 1-methylcyclopropene (1-MCP), ethylene (either pre- or post-inoculation) or aminoethoxyvinylglycyne (AVG). Values are presented as the average ± standard error (*n* = 4). Significant differences, determined by ANOVA and Fisher’s least significant difference test with *p* ≤ 0.05, are indicated by lower case (for endophytic populations) or upper case (for epiphytic populations) letters.

Psa strains producing ethylene induced stomata opening in the host plants and showed a faster endophytic colonisation. Moreover, Psa infection induced ethylene emission in the host plant and promoted the expression of genes related to ethylene signal cascade. To investigate the overall effect of ethylene on the onset of infection, in absence of kiwifruit mutant for the ethylene metabolism or perception, the host plants were treated with inhibitors of the ethylene biosynthesis (AVG) or perception (1-MCP), or with ethylene. Moreover, ethylene was applied either 3 days before inoculation (pre), or 3 days before and immediately after inoculation (post), the latter treatment to mimic the bacterial contribution to the emission of ethylene. 1-MCP and ethylene pretreatments had no significant effect on endophytic Psa populations. In contrast, ethylene treatment immediately after inoculation and AVG promoted endophytic bacterial growth (Figure 11). For the duration of the experiment, no symptoms could be specifically attributed to Psa.

## 3. Discussion

### 3.1. Ethylene Emission by Pseudomonas syringae pv. actinidiae

Ethylene production was detected from Psa strains belonging to biovar 3 (CFBP7286, 10787, Arg2.1), but not from strains belonging to biovar 1 (NIAS302143, NIAS302145, SUPP319, SUPP320) or 2 (Psa-K2, KACC10594). The highly virulent biovar 3 strains are responsible for the pandemics started in 2008, whereas strains from other biovars, although able to cause disease, raise a moderate sanitary concern [18,19]. Thus, the ability to produce ethylene may contribute to Psa virulence, as it does for other *P. syringae* pathovars, such as pvs. *glycinea* and *phaseolicola* [9,39], as well as for *Ralstonia solanacearum* strains [40,41]. In the latter species, ethylene production is coregulated with the type 3 secretion system, suggesting its function in pathogenesis.

However, no molecular or biochemical evidence was obtained in Psa for the 2-oxoglutarate-dependent ethylene-forming enzyme (EFE), which is a crucial enzyme in ethylene biosynthesis for other *P. syringae* pathovars and *R. solanacearum* strains [9,40].

In contrast, the genome comparison between strains of different biovars allowed the identification of a sequence (*bep*), specific to biovar 3 and putatively encoding for a member of the Fe(II)/2-oxoglutarate hydroxylase family. This family includes all the ethylene-producing enzymatic activities characterised so far: the plant, 1-aminocyclopropanecarboxylic acid-dependent one, and the two bacterial reactions proceeding from KMBA and from 2-oxoglutarate [34]. The role of *bep* in ethylene biosynthesis was confirmed by site-specific mutagenesis, since ethylene emission was abolished in defective mutant (CFBP7286-Δ*bep*). Therefore, bep enzyme may catalyse the oxidative decarboxylation of a yet unidentified compound (or an array of molecules with similar characteristics), ultimately yielding ethylene. However, the low ethylene emission levels, compared to those of *P. syringae* pv. *glycinea*, suggest that the reaction efficiency may be low, or its substrates/cofactors may be limiting. The contribution of plant metabolism to overall ethylene levels (Figure 9), possibly with a feedback stimulation after the bacterial trigger, may compensate for the low production efficiency by Psa.

### 3.2. Modulation of Ethylene Emission in Pseudomonas syringae pv. actinidiae

Ethylene emission by Psa strains belonging to biovar 3 was observed only in FPE medium, indicating that one or more heat-stable plant derived compounds, with a molecular weight below 10 kDa, are required for its production. Therefore, the fraction of the plant extract below 10 kDa may contain either an essential substrate or cofactor for the production or a molecule acting as a signal for the perception of the host by Psa.

The dark-conditioned ethylene production from infected microcuttings (Figure 8) may be explained by some biochemical consequences of photosynthetic inactivity. The addition of reduced or oxidised glutathione to Psa cultures in plant extract, respectively, abolished or permitted ethylene production (Figure 3), in agreement with the predicted plant cell redox status. In fact, reducing potential (NADPH, reduced ferredoxin and thioredoxin) is generated as the end product of the photosynthetic electron transport chain. Thus, the plant redox status is a likely determinant of ethylene emission from Psa. Notably, the key mediator of SA-dependent responses, NPR1, requires reduction to be converted to the monomeric, active form. Thus, Psa-dependent ethylene emission and NPR1 activity take place in opposite redox conditions. A speculative explanation of the role of dark-conditioned ethylene production by Psa in the apoplast may reside in the circadian cycle of SA synthesis, which takes place by night [42]. In this sense, bacterial ethylene release might quench SA signalling before the activation of defence responses. Psa may as well promote its own infection process with a feedback mechanism based on ethylene release, i.e., the endophytic population would facilitate the migration into the apoplast of epiphytic bacteria by preventing stomata closure or forcing stomata opening. Additionally, endophytically growing Psa would benefit from an ethylene-stimulated, incremented availability of nutritional resources, such as soluble sugars [43,44] or iron [45,46], which are stored in the chloroplast and released into the apoplast during photosynthetic inactivity.

Both plant- and Psa-derived ethylene seem to concur to overall ethylene release. In fact, the inclusion of AVG in plant growth medium reduces overall ethylene emissions, but Psa-infected samples withhold a higher emission (Figure 9), which may be explained assuming that the bacterial ethylene synthesis pathway is not affected by AVG.

### 3.3. Perception of Exogenous Ethylene by Psa

The perception of ethylene by plant-associated bacteria has been studied in a few species. Positive chemotaxis toward ethylene has been previously shown for several *Pseudomonas* species [47]. The adaptive function underlying an ethylene perception system in pathogenic bacteria may reside in the ability to localise a pervious entry point, such as stomata or open wounds (by the emission of host-derived ethylene). Another example of ethylene perception in bacteria is provided by the fruit coloniser *Komagataeibacter xylinus*, in which ethylene associated to fruit ripening enhances biofilm formation and facilitates epicarp colonisation [48]. In contrast, the reduced virulence of *Agrobacterium tumefaciens* induced by ethylene is probably mediated by metabolic changes in the host, rather than directly elicited in the pathogen [49], suggesting that ethylene perception is not universally spread in bacteria, and is the probable consequence of functional adaptation.

The ability of Psa to perceive ethylene is demonstrated by the induction of motility and the expression of virulence genes (Figure 4). While flagella do not seem implicated in ethylene-induced motility, genes governing the assembly (*pilC*) and function (*pilO*) of pili respond to ethylene. In addition to bacterial motility and exploration, pili play a role in biofilm formation, surface adhesion and cell-to-cell communication [8], thus potentially contributing to multiple aspects of bacterial virulence.

In alternative, ethylene perception by Psa might amplify the activation of virulence factors as a signal of a successful host–pathogen recognition, whatever its source (either the plant or the bacterium) might be. In fact, ethylene is per se able to activate virulence-related genes encoding for type 3 effectors (*avrPto1*, *hopD1*, *hopR1*) (Figure 4), even in absence of more host-specific cues.

### 3.4. Regulation of Stomata Opening by the Plant and the Pathogen

Ethylene plays an important role in the pathogen penetration into the plant tissues since it may induce stomata opening and promote bacterial motility, thus facilitating host invasion. Part of the pathogenetic cycle of Psa takes place in the phyllosphere, with no disease symptoms associated [23], and stomata are strong candidates as the main entry point [25]. This view agrees with the early observation that bacterial canker is promoted by high relative humidity [50], which could provide a water film for the bacterial migration and allow stomata opening. In addition, after host invasion, (hemi)biotrophic pathogens such as Psa could take advantage in promoting leaf carbon fixation by forcing stomata opening.

Both the host and the pathogen concur to stomata opening regulation. In fact, the coronatine-producing Psa-K2 strain increased stomata opening in *A. chinensis* regardless of ABA treatment (Figure 6d and Figure 7b), while the ethylene producing strain, CFBP7286, induced a moderate opening which was abolished by ABA (Figure 6d and Figure 7b). ABA treatment did not contrast coronatine effects, in agreement with previous observations [3]. The increase of stomata opening was transient, suggesting a plant response after pathogen recognition. Interestingly, in *A. deliciosa*, which is substantially more resistant to Psa than *A. chinensis* [18,51], the presence of the pathogen induced a clear stomata closure. Stomata closure was higher in CFBP7286- than in Psa-K2-inoculated plants, suggesting that the production of coronatine may at least partially counteract the plant reaction to the pathogen (Figure 6c). Thus, species-dependent sensitivity to coronatine and ethylene and immediacy of pathogen recognition may be related to the higher susceptibility of *A. chinensis* to bacterial canker, in comparison to *A. deliciosa* [51].

### 3.5. Role of Ethylene in the Host–Pathogen Interactions

Ethylene has multifaceted effects on the interaction between the host plant and Psa, being both a virulence factor and a key hormone regulating plant defences. As a virulence factor, ethylene may promote stomata opening, thus facilitating the penetration into the host tissues, and, at the same time, it may reduce the host defences by silencing SA-mediated signalling and helping Psa to escape the hypersensitive response [52,53]. In agreement with this view, *A. deliciosa* defences could be successfully stimulated in previous work [30] by applying SA or its functional homologue, acibenzolar-S-methyl, whereas methyl-JA treatment promoted ethylene production/signalling and Psa growth in plant tissues [54]. In addition, gene repression of *ics1*, which encodes for the precursor enzyme of SA biosynthesis (isochorismate synthase), and of SA-dependent responses (*PR* genes) was reported after infection of *A. deliciosa* with Psa strain CFBP7286, while the same genes were promoted when uninfected plants were exposed from volatile compounds emitted by infected plants [55]. The direct production of ethylene by *P. syringae* pathovars [39] and the HopAB2_PtoDC3000_-mediated induction of ethylene synthesis from inoculated plants [14] were previously observed to contribute to bacterial virulence. In this work, both the pathogen and the host seemingly concur to ethylene production.

In agreement with previous transcriptomic studies [56], the activation of the ethylene signalling pathway in inoculated plants is detectable as soon as 6 h post inoculation (Figure 10) and requires the pathogen’s ability to synthesise ethylene. Although both *Actinidia* species show a similar trend, the stronger induction in *A. chinensis* may underlie its higher susceptibility to Psa in comparison to *A. deliciosa*. EIN3, which integrates ethylene and JA signalling [57] and prominently contributes to SA silencing by inhibiting *ics1* transcription in *Arabidopsis* [58], may be a key factor for the overcoming of SA-mediated defences by pathogens.

Since Psa endophytic growth is only partially affected by ethylene pretreatment or 1-MCP, compared to untreated plants, ethylene appears to be an accessory virulence factor, unlike in *P. syringae* pv. *glycinea*. However, the deletion of *bep* causes a significant reduction of growth rate in planta (Figure 5). Thus, the capability to produce ethylene may be a recently evolved feature that enhanced the virulence of Psa in its arms race against the host, rather than an essential condition for host compatibility.

Since plant pretreatments with AVG and exogenous ethylene, respectively, promote or reduce endophytic Psa colonisation, it can be concluded that plant ethylene production, in the early phases of plant defences, counteracts pathogen infection or concurs to defence priming, which is an efficient mechanism for increasing defences against multiple phytopathogens [59]. In contrast, the application of AVG, by inhibiting ethylene production, prevents this process. Besides, a further ethylene application following infection may, on the one hand, hijack plant defences suppressing the SA-mediated responses, and, on the other hand, stimulate pathogen virulence by regulating pathogenicity effector genes.

### 3.6. Conclusions

The present work aimed to demonstrate the capability of Psa to produce ethylene, and to investigate its possible roles in pathogenicity. The first evidence of ethylene emission from different Psa strains is provided here, along with the molecular basis of its biosynthesis and regulation. Only the highly virulent strains, belonging to biovar 3, can produce ethylene and induce its synthesis in infected plants. In the attempt to elucidate the genetic base of ethylene production, *bep* has been identified as the likely gene responsible for this trait.

By the data collected in this work, several ecological roles may be postulated for ethylene in the interaction between by Psa and its *Actinidia* spp. host. As a biotrophic pathogen, Psa may take advantage from an ethylene-induced, senescence-like syndrome, with the demolition of plant cell storage compounds and the release of simple nutrients (mono- or disaccharides, aminoacids, minerals) into the apoplast. Additionally, ethylene-releasing strains enhanced stomata opening, and exogenous ethylene induced virulence and motility genes in Psa.

Taken together, these observations support a role of ethylene as virulence factor. However, Psa biovars unable to produce ethylene retain their pathogenic potential. Thus, the contribution of ethylene to Psa virulence is probably accessory. A better understanding of the relation between ethylene manipulation and bacterial virulence may come from the recently identified Psa biovars 5 and 6 [21,22], for which molecular data were not available so far.

## 4. Materials and Methods

### 4.1. Bacterial Strains, Culture and Inoculation

The list of bacterial strains employed in the experiments is reported in Table 1. The medium used for the experiments (Fresh Plant Extract, FPE) was obtained by homogenising in vitro, axenic micropropagated *Actinidia chinensis* var. *deliciosa* ‘Hayward’ leaves (30 g FW L^−1^) in 10 mM phosphate buffer saline, pH 7.0, containing polyvinylpolypyrrolidone (1% *w/v*). After centrifugation (10,000× *g* for 20 min), the supernatant was sterilised by filtration through a Millipore (Merck KGaA, Darmstadt, Germany) filter (0.22 μm) and used within 24 h.

The inocula were produced from bacterial cultures, grown on FPE for 2 days at 27 °C under moderate agitation (80 rpm) and resuspended in 10 mM MgSO_4_ to a final concentration of 10^8^ colony forming units (CFU) mL^−1^. Sterile MgSO_4_ was used for mock-inoculation. The inoculation of in vitro plantlets was performed by dipping each plant for 10 s in the bacterial suspension.

For all the experiments, bacterial titres were determined by producing serial 1:10 dilutions of the bacterial suspensions, and plating 10-μL drops of each dilution on LA medium.

### 4.2. Plant Material and Growing Conditions

In vitro cultivated plantlets of *A. chinensis* var. *deliciosa* ‘Hayward’ and *A. chinensis* var. *chinensis* ‘Hort16A’ (indicated as *A. deliciosa* and *A. chinensis*, respectively), provided by Vitroplant Italia Srl (Cesena, Italy) were propagated and rooted in Murashige-Skoog (MS) medium. Two weeks before the experiments, plants were singularly transferred to glass vials (150 mL volume) containing 20 mL half-concentration MS mineral salts medium, adjusted to pH 5.7. Plants were kept in a growing chamber at (22 ± 2) °C, with a 16:8 h light-dark period.

Two- or three-year old potted plants of the same cultivars were maintained in greenhouse conditions (approx. 65% relative humidity, 24 °C). The growing medium was obtained by mixing 1:1 (*v*/*v*) peat and sand, with standard drip irrigation (1.33 L day^−1^ plant^−1^). The peat mineral concentration declared by the manufacturer was: NH_4_^+^ 25 g m^−3^, NO_3_^−^ 35 g m^−3^; P_2_O_5_ 104 g m^−3^, K_2_O 120 g m^−3^; MgO 12 g m^−3^; micronutrients 25 g m^−3^.

### 4.3. Ethylene Measure Systems

Three different apparatuses for the quantitative detection of ethylene were employed. A DANI HT 86.01 gas chromatograph (DANI Analitica, Milan, Italy) equipped with and a Porapak Q column (Supelco, Bellefonte, PA, USA) at 80 °C under a nitrogen flow (16 mL min^−1^), and a flame ionisation detector at 150 °C, was used on bacterial cultures for static headspace measurement of relatively high (>100 nL L^−1^) ethylene concentrations. However, a higher sensitivity and a lower detection limit (at nL L^−1^ level) were required to study physiological processes involving ethylene. In particular, prolonged headspace preconcentration of ethylene, necessary to achieve a better sensitivity and a more efficient GC measurement, should be avoided [60]. For more sensitive applications and/or shorter headspace accumulation analyses, a PTR-ToF-MS 8000 apparatus (Ionicon Analytik GmbH, Innsbruck, Austria) equipped with a system allowing for primary ion switching (in this case O^2+^ mode), with drift tube setting at 110 °C, 2.25 mbar and 550 V, was employed to detect masses related with the chemical ionisation of ethylene, such as fragments of *m/z* = 26.0158, 27.0260, 28.0314, 29.0155 and 42.0125 [61]. Ethylene readings obtained with this method were validated by comparison with gas-chromatographic data. Finally, real-time emissions were monitored by using a laser-photoacoustic detector (ETD-300, Sensor Sense, Nijmegen, NL). The application of this detector in combination with a flow-through system was proven to be more performing in sensitivity and time response in comparison to gas chromatography [62]. Briefly, the laser-based ethylene detector consists of a CO_2_ laser and a photoacoustic cell, in which the gas is detected. Traces of ethylene released by biological samples can absorb the laser radiation inside the photoacoustic cell; the absorbed energy is released as heat, which will create a pressure increase inside a closed volume. By modulating the laser at an acoustic frequency, a periodic pressure wave is generated that can be detected with a sensitive miniature microphone.

### 4.4. Ethylene Emission by Pseudomonas syringae pv. actinidiae

Cultures of the different Psa strains, belonging to the biovars of highest phytosanitary concern (1, 2 and 3), were grown in 50 mL air-tight vials, containing 5 mL of FPE. Ethylene emission by bacteria grown in artificial medium was also assessed. Ceria 132 minimal medium amended with Murashige-Skoog vitamins was used to mimic the oligotrophic conditions of FPE. Vials were incubated in the dark at 27 °C under moderate shaking (80 rpm) to stationary phase (3 days). Ethylene levels were determined by gas chromatography or PTR-ToF-MS. *P. syringae* pv. *glycinea* was included in the experiment as the positive control [9]. For each of the strains, three replicates of three vials were analysed. Readings taken from non-inoculated FPE were assumed as the baseline and subtracted from all other measures. Each strain was tested on three independent experiments.

Ethylene production kinetics during bacterial growth was monitored in Psa biovar 3 strain CFBP7286. The bacterium was inoculated in air-proof 500 mL vials closed with a butylene stopper, containing 50 mL of FPE and maintained for 3 days at 27 °C under shaking. Every 8 h, headspace ethylene concentration was measured, and a 1-mL aliquot of the medium was taken with a syringe to determine the bacterial population. Vials containing axenic medium were included for reference.

### 4.5. Biochemical and Molecular Basis of Ethylene Emission by Pseudomonas syringae pv. actinidiae

To understand the biochemical basis of ethylene emission, cultures of Psa strain CFBP7286 in FPE were amended with the precursors of described biosynthetic pathways, i.e., L-methionine (1 and 0.01 mM), or 2-oxoglutarate (10 and 0.1 mM), before measuring ethylene emissions as previously described. In all these experiments, the baseline was determined with the respective non-inoculated, modified FPE.

Moreover, the presence of the genes of the key enzymes for the two pathways was verified in the genomes of different Psa strains (NCPPB3871 and PA459, belonging to biovar 1, and CFBP7286, belonging to biovar 3) deposited in the NCBI database. BLAST alignment of these genotypes was attempted to identify sequences present in biovar 3, but not in biovar 1 genomes.

PCR was subsequently performed on the Psa strains listed in Table 1 to test for presence of *efe* and of the newly identified Bacterial Ethylene Putative Producer (*bep*) in each of them, using the primers shown in Table S1.

The suicide vector pKnock-Km [63] was applied to Psa strain CFBP7286 to produce a *bep*-defective mutant (CFBP7286-Δ*bep*) by site-specific mutagenesis. The success of mutagenesis was checked by end-point PCR and quantitative PCR as described in Section 4.7. The abolishment of ethylene production by CFBP7286-Δ*bep* was assessed by gas chromatography and PTR-ToF-MS.

### 4.6. Modulation of Ethylene Emission in Pseudomonas syringae pv. actinidiae

Since ethylene emission by in vitro culture of Psa biovar 3 was recorded only when the pathogen was grown in plant extract medium, but not in artificial medium, experiments aiming at determining which fraction of plant extract primed the bacterial ethylene production were performed. In one test, FPE was boiled for 5 min before inoculation to denature plant proteins and prevent any enzymatic activity inside the medium. In a second experiment, FPE was separated by size exclusion chromatography into three fractions, including the compounds with molecular weight above 30 kDa, between 10 and 30 kDa, and below 10 kDa. The fractions were separately added to an artificial medium (Ceria 132, amended with 30 g L^−1^ sucrose, 100 mg L^−1^ myo-inositol, 1 mg L^−1^ thiamine-HCl, 1 mg L^−1^ nicotinic acid, 1 mg L^−1^ pyridoxine, 1 mg L^−1^ glycine) with negligible basal ethylene emission. Ethylene release was measured from the resulting medium after Psa CFBP7286 growth for 48 h.

Since ethylene emission from infected plants was recorded only by night, the bacterial sensitivity to light was tested. In order to do so, Psa CFBP7286 was grown in FPE either in the dark, or under a continuous lighting. Moreover, since light conditions influence the plant cell redox state (which is reducing in photosynthetically active plants, and oxidising in the dark), the FPE medium was amended with either a reductant (2 mM reduced glutathione, GSH) or an oxidising substance (1 mM oxidised glutathione, GSSG), to simulate bacterial growth in planta during light or dark, respectively. Other reductants (NADH, ascorbate, dithiothreitol) or oxidisers (NAD^+^, dehydroascorbate) were also tested at 2 mM. Each condition was tested in two independent experiments.

### 4.7. Effect of Ethylene on Bacterial Growth, Motility, Virulence and Host Plant Colonisation

To assess the importance of ethylene in host plant colonisation by Psa, in absence of kiwifruit mutants impaired either on ethylene production or perception, a biochemical interference on *A. deliciosa* ethylene metabolism was adopted. For this purpose, *A. deliciosa* plants were treated with the inhibitor of ethylene biosynthesis in plants, aminoethoxyvinylglycine (AVG), with the inhibitor of ethylene perception, 1-methylcyclopropene (1-MCP; commercial formulate: AFxRD-0014, Rohm and Haas, Philadelphia, PA, USA), or with ethylene. AVG (1 g L^−1^ active ingredient) was included in the plant growing medium. Treatments with 1-MCP (20 µL L^−1^) were performed by including 60 mg of formulate in a 500 mL-pot containing the sample plants, and pouring water on it through a pierceable septum. Ethylene treatments were done by releasing ethylene from 10 µL 2-chloroethylphosphonic acid (ethephon) in KOH in a 1-L jar, determining its concentration by gas chromatography, and transferring an appropriate headspace volume to the growing vials with a syringe. As no correlation was observed between plant colonisation by Psa and ethylene treatment concentration in the range between 0.5 and 50 µL L^−1^, all further analyses were performed with 1 µL L^−1^ ethylene. To determine the influence of treatment timing with respect to inoculation, ethylene was administered once, immediately post-inoculation, or twice (3 days pre-inoculation + immediately after inoculation). Each treatment was independently replicated at least three times and included 4–6 plants. Plants were inoculated with the Psa strain CFBP7286-GFPuv, belonging to biovar 3. To monitor bacterial colonisation of the host plant, 7 days after inoculation, epiphytic and endophytic bacterial populations were determined. The plants were washed for 30 min in 10 mM MgSO_4_ in an orbital shaker (120 rpm). Subsequently, they were externally sterilised with successive washes in 70% ethanol and 1.5% NaClO, rinsed twice in sterile water, and homogenised in 10 mM MgSO_4_. The bacterial population was determined in the external wash and in the homogenate, representing the epiphytic and endophytic populations, respectively.

The effect of ethylene on bacterial growth was assessed on LB cultures, with a headspace ethylene concentration of 1 µL L^−1^. The motility phenotype of Psa was assessed on LB medium containing 0.4% (*w/v*) agar. After inoculation, the plates were enclosed in an air-tight 1.5 L jar, with a headspace concentration of 1 µL L^−1^ ethylene. Seven days later, the percentage of cultures showing a distinct motility phenotype was determined and compared to plates not exposed to ethylene.

The expression of genes involved in bacterial motility and virulence was tested in Psa strain CFBP7286, grown for 3 days in liquid LB medium in a 500-mL jar containing 1 µL L^−1^ ethylene. After the precipitation of bacteria, RNA was extracted using Total RNA purification kit (Norgen Biotek, Thorold, ON, Canada). After a double treatment with DNase (Sigma-Aldrich, St. Louis, MI, USA), RNA was quantified by spectrophotometric analysis, checked by gel electrophoresis, and retrotranscribed to cDNA using the High Capacity cDNA synthesis kit (Life Technologies, Rockville, MD, USA). Quantitative PCR analyses were performed on a StepOne Plus machinery (Applied Biosystems, Foster City, CA, USA) using the Power SYBR Green chemistry (Life Technologies). The primers (Table S1) were designed using Primer3plus (https://primer3plus.com/cgi-bin/dev/primer3plus.cgi) (accessed on 9 January 2017) [64] and checked for specificity by melting curve analysis and endpoint PCR, using 50 ng of genomic DNA as the template. The following PCR program was followed: 1 cycle at 50 °C for 2 min, 1 cycle at 95 °C for 10 min, 40 cycles at 95 °C for 15 s and 61 °C for 1 min. Gene expression was normalised to *recA* and *rpoD* [65,66,67] and quantified through the comparative C_t_ method [68].

Finally, the effect of Psa infection on stomata opening was studied. Stomata opening was evaluated on *A. deliciosa* and *A. chinensis* potted plants, sprayed with sterile water or a water suspension of Psa strains SUPP319 (biovar 1), Psa-K2 (biovar 2) or CFBP7286-GFPuv (biovar 3). To induce stomata closure, 1 day prior to inoculation, a subset of plants was sprayed with 0.5 mM ABA. Control plants were mock-treated with water. Stomata opening was assessed both by direct microscopy measurement or by assessing stomata conductance. Direct measuring was performed using a Nikon C1-S confocal laser scanning microscope (Nikon Instruments Corporation, Tokyo, Japan) equipped with an Argon laser [56]. Optical sections of leaf lamina were acquired at 40, 60 and 100× with Nikon PlanApo (Nikon Instruments Corporation) objectives and the BHS (GHS) filter set. Images were acquired and analysed by the NIS-Elements C Microscope Imaging Software (Nikon Instruments Corporation). Stomatal conductance and gas exchange were measured by means of a CIRAS-1 equipment (PP Systems, Amesbury, MA, USA). Readings were taken at 24 (just before ABA treatment) and 20 h before inoculation, just before inoculation (0 h), 4 and 24 h after inoculation. The experiment was performed three times independently, and each treatment included four plants. On each plant, the first three fully expanded leaves were considered for the measurements.

### 4.8. Ethylene Emissions from Infected Plants

The bacterial strains used for this experiment are listed in Table 1. In a first experimental setup (independently replicated three times), *A. deliciosa* plantlets were inoculated with Psa strain CFBP7286 and *P. syringae* pv. *syringae* strains. Subsequently, the experiment was performed with strains of *P. syringae* pv. *actinidiae* belonging to different biovars. Ethylene emissions were monitored for 3–4 days by laser-photoacoustic detection in real time, or by PTR-ToF-MS after an 8-h accumulation of headspace. Non-inoculated plants were included in the experiment as negative controls, and emission from axenic medium were considered as baseline signal. To determine the bacterial contribution of ethylene release, the plants were transplanted to an AVG-containing medium (1 g L^−1^ active ingredient). Part of the plants was inoculated with CFBP7286 3 days after transplant. Ethylene concentration was determined by gas chromatography after 1 week of headspace accumulation.

To correlate ethylene emission with the bacterial population *in planta*, after ethylene monitoring, epiphytic and endophytic bacterial populations were determined as previously described.

### 4.9. Effect of Psa Infection on Ethylene Signalling in Host Plants

The induction of genes coding for ethylene receptors, signal transducers and transcription-activating factors was tested on *A. chinensis* and *A. deliciosa* plantlets inoculated with CFBP7286. Positive controls were represented by *A. deliciosa* plants exposed to 1 µL L^−1^ ethylene. Mock-inoculated plants were included for reference.

Six hours post inoculation, RNA from plant samples was extracted and purified using Total RNA purification kit (Norgen Biotek). Quantitative PCR analyses were performed as described in Section 4.7. The reactions, performed in triplicate, were incubated 2 min at 50 °C and 5 min at 95 °C, followed by 40 cycles of 95 °C for 15 s and 60 °C for 1 min, with data collection at each annealing step. Gene expression was calculated by the comparative 2^−ΔΔCt^ method [69,70] normalised to glyceraldehyde-3-phosphate dehydrogenase (*GAPDH*) and actin as the housekeeping genes [71].

### 4.10. Statistical Analysis

Student’s T test was used for pairwise comparisons. ANOVA and Fisher’s least significant difference test were applied to multiple comparison of data sets. Statistical computation was performed using the Statistica software (ver. 5.0, Statsoft Inc., 1995, Tulsa, OK, USA).

## Figures and Tables

**Table 1:** List of the *Pseudomonas syringae* pathovars and strains used in this work.

Strain	Biovar	Origin	Source or Reference
*Pseudomonas syringae* pv. *actinidiae*			
SUPP319	1	Japan	[35]
SUPP320	1	Japan	[35]
NIAS302143	1	Japan	NIAS, National Agriculture and Food Research Organization (Japan)
NIAS302145	1	Japan	NIAS, National Agriculture and Food Research Organization (Japan)
KACC10594	2	Korea	KACC, National Institute of Agricultural Sciences (South Korea)
Psa-K2	2	Korea	[36]
CFBP7286	3	Italy	CIRM-CFBP, INRA (France)
CFBP7286-GFPuv	3		[25]
CFBP7286-Δ*bep*	3		This work
10787	3	New Zealand	[37]
Arg2.1	3	Argentina	[38]
*Pseudomonas syringae* pv. *syringae*			
ICMP3523		Australia	ICMP, Landcare Research (New Zealand)
LT23		Italy	JL Vanneste, direct isolation from *A. deliciosa*
*Pseudomonas syringae* pv. *glycinea*			
NCPPB1883		USA	NCPPB, FERA (UK)

## Supplementary Materials

The following are available online at https://www.mdpi.com/article/10.3390/ijms22094375/s1.
