# Supplementary material for: A Breach in Plant Defences: Pseudomonas syringae pv. actinidiae Targets Ethylene Signalling to Overcome Actinidia chinensis Pathogen Responses
Source: Int J Mol Sci. 2021 Apr 22;22(9):4375. doi: 10.3390/ijms22094375 (PMC8122719; doi:10.3390/ijms22094375)
Supplement: Supplementary file 1 [file ijms-22-04375-s001.zip › ijms-1167362-SI.pdf]

Supporting information

Article

A breach in plant defences: *Pseudomonas syringae* pv. *actinidiae* targets ethylene signalling to overcome *Actinidia chinensis* pathogen responses

Antonio Cellini [1], Irene Donati [1], Brian Farneti [2], Iuliia Khomenko [2], Giampaolo Buriani [1], Franco Biasioli [2], Simona M. Cristescu [3] and Francesco Spinelli [1,*]

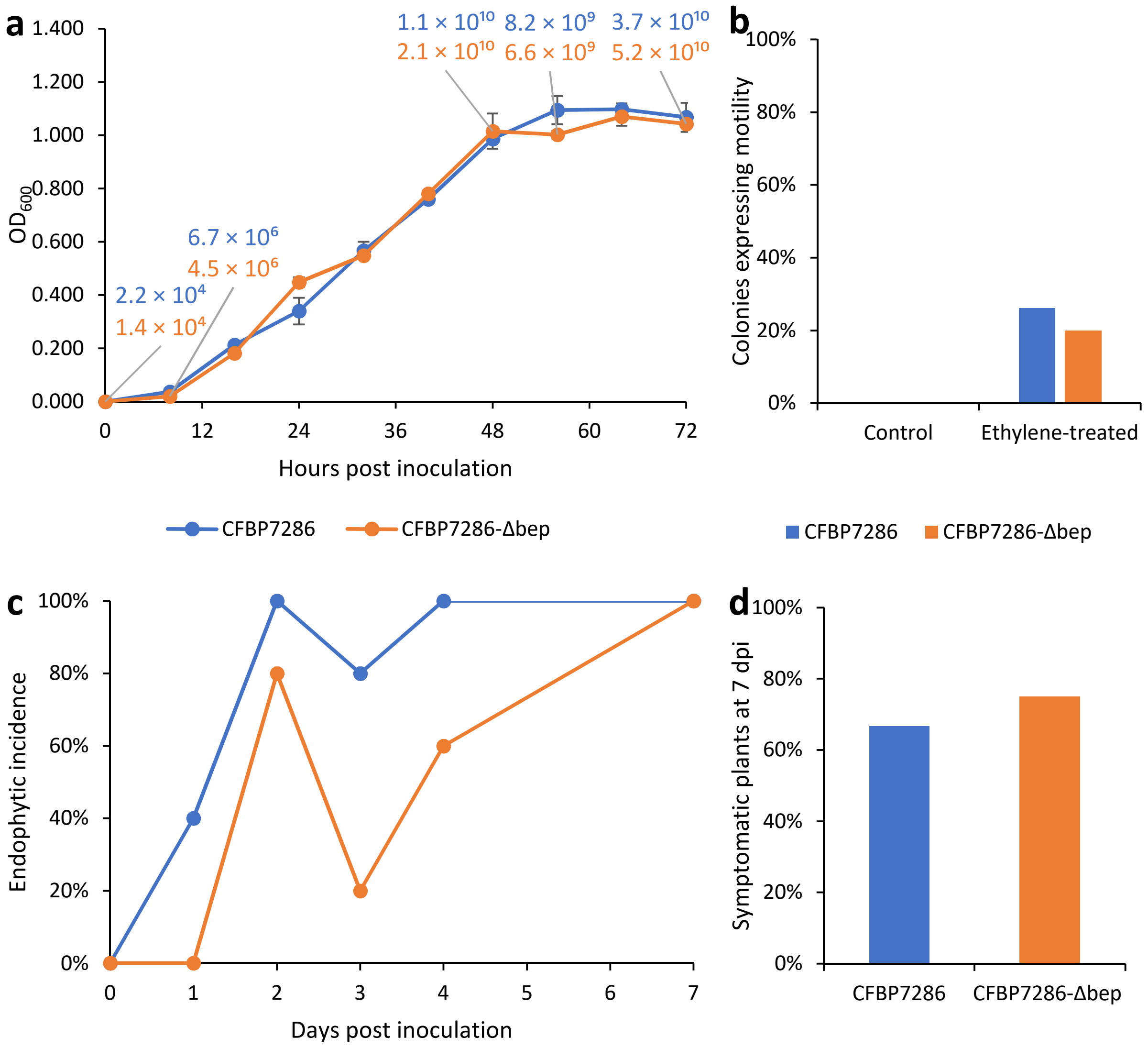

Figure S1. Comparison between *Pseudomonas syringae* pv. *actinidiae* wild-type strain CFBP7286 and *bep*-defective (Δ*bep*) mutant. (**a**) Growth monitored in LB medium during 72 h. (**b**) Percentage of colonies expressing a motility phenotype under ambient air (Control) or 1 μL L^{-1} ethylene (Ethylene-treated). (**c**) Incidence of endophytic bacterial population in experimentally inoculated *Actinidia deliciosa in vitro*

microexplants, and (**d**) percentage of plants showing a symptomatology (apical necrosis and/or leaf pedicel browning) 7 d post inoculation.

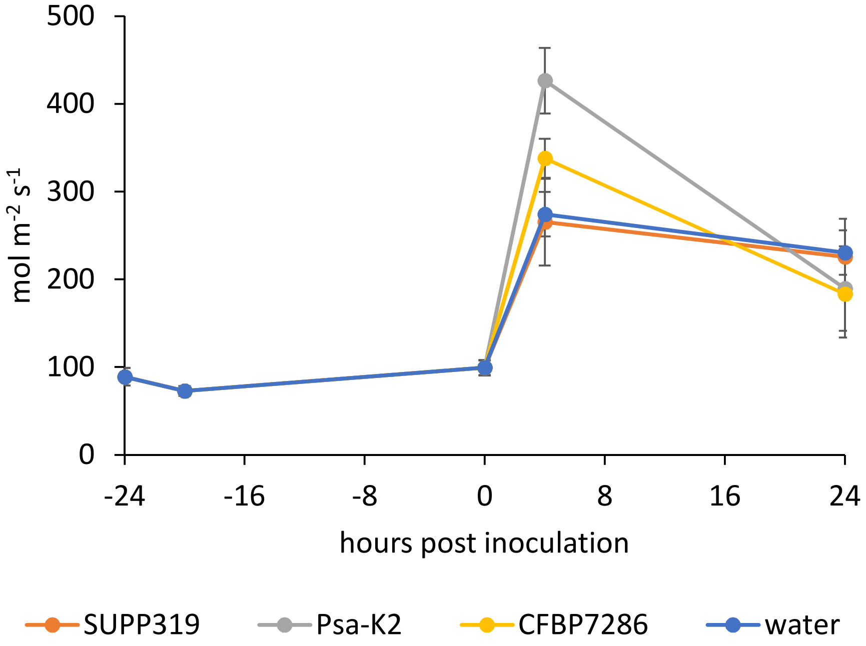

Figure S2. Stomatal conductance of *Actinidia chinensis* plants after inoculation with *Pseudomonas syringae* pv. *actinidiae* strains SUPP319 (biovar 1), Psa-K2 (biovar 2) or CFBP7286 (biovar 3), measured in real-time by gas exchange analyser (CIRAS-1). Data are presented as the average ± standard error (n = 3).

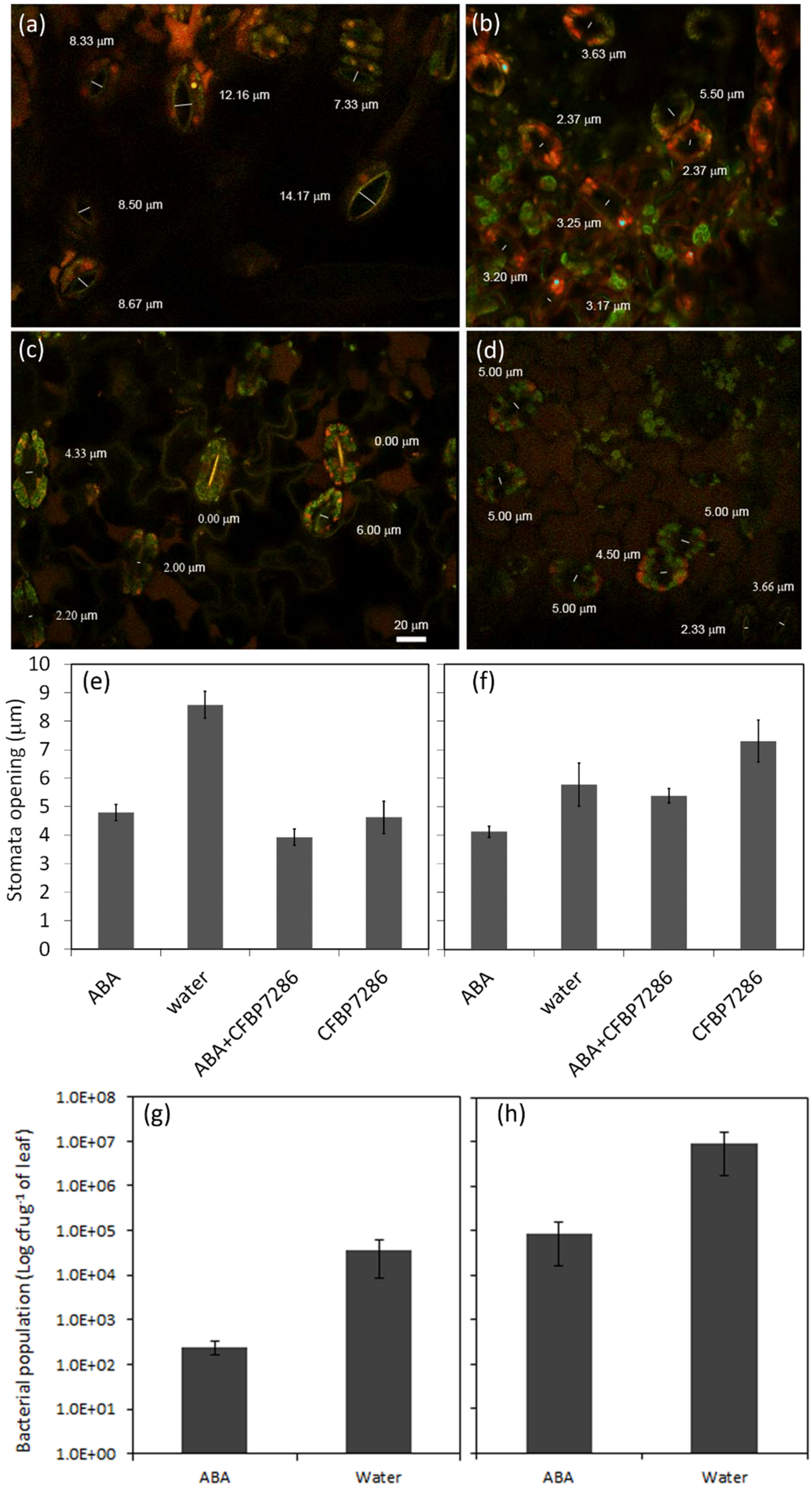

Figure S3. Confocal laser scanning (CLSM) micrographs showing stomata of *Actinidia deliciosa in vitro* plants (**a-d**). Stomata opening was measured via Nikon, NIS-Element software. CLSM micrographs were taken 24 hours after inoculation with *Pseudomonas syringae* pv. *actinidiae* (Psa) CFBP7286-GFPuv and 48 hours after ABA application. Inoculation was performed by manually spreading the bacterial suspension on the left hand of

kiwifruit leaf lamina. Stomata opening was measured on the right hand of the lamina to verify the effect of a diffusible signal. (**a**) Water, (**b**) CFBP7286-GFPuv, (**c**) ABA, (**d**) ABA + CFBP7286-GFPuv. (**e-f**) effect of ABA application and/or CFBP7286-GFPuv inoculation on stomata opening in (**e**) *Actinidia deliciosa* or (**f**) *Actinidia chinensis*. (**g-h**) Endophytic population of Psa CFBP7286-GFPuv in *Actinidia chinensis* leaves treated with ABA or water 48 hours before inoculation (**g**) 3 or (**h**) 7 days after inoculation. In (**e-h**), data are presented as the average ± standard error (n = 4).

Table S1. List of primers used for gene expression studies and bacterial genome surveys.

Gene	Primer F	Primer R
actin	CCAAGGCCAACAGAGAGAAG	GACGGAGGATAGCATGAGGA
aco	GGAAGTGTTGACTAACGGAAGG	TCGCCCACCAGATTGTAGAA
acs	CTGGCATTCTGTTTGGCTGAC	GTGTTCCTGTTCTCCATCTCAAGT
avrpto1	GGAGCGAATCTTGCCATT	GGAGCGATATGCGTGAAG
bep	TCGTCTGCTTGACGAACTTG	AATCGCTGGTGTTCTTCCAC
efe	TCATGAGCCTGTCGCGCG	TAAGCCACCAACATCGTC
ein2	TGGTAGAGATGATGTTTGGGAACT	CAGAGCAAGGTAGAAGCAGTGA
ein3	GGACAGTGACAGCGACTATGAC	GGTTGGCTAGACGGTTCTACATC
enolase	CATCGCCAACCTCAATGG	CCTGGATGTCGATGTTGTTAT
erf1	TCCAAGATGAAGAGACCGAAGTGA	GCCGTCAAGATAAGGGATCTGGTA
erf2	CCTTCATCTCCCACAACCA	AACTGTCAGAAGTCGAGGAA
erf3	CAAAGGCGATGTATGGTCCAAGTG	CCGATGTAGTAGGCAGTGAAGCA
erf4	CGGTAGTTTCTCGCTTCTCTC	ACGGACGCTGCCTAATTC
etr1	GCTGGAGCTTGTCGAAGTAGTTG	GAAGATCCCTAGCCCTCATTGACT
fliP	TCAAGACGGCGTTTCAGA	CGGCGAGAGCATCATCAT
GAPDH	GTTCCCACTGTCGATGTCTCA	CCCTTCATCTTGCCCTCAGA
hopD1	CAGTAGACAGCAGTAGCC	CGGGTTATCGGAAACAAG
hopS2	CCTTAAACGGCTGGCAGAG	CGAAGTGATGCTTGAGGTGAA
hopZ5	TCAGGCTACAATACTTACGCATCA	CAGGAATAGAACGGAACTCAGGAT
pilA	GCCATTCCTTCCTATCAA	GTAAGACCATTGCTCCAG
pilC	CGCTGGACATCGCATTCT	GCACCTTCGGCAATGATG
pilO	CCTACAGAAAGCAGATGGA	GTGATGTCTTCAAGCAGTC

recA	CGCACTTGATCCTGAATACG	CATGTCGGTGATTTCCAGTG
rpoD	CCGAGATCAAGGACATCAAC	GAGATCACCAGACGCAAGTT
